# Impact of red cell distribution width and red cell distribution width/albumin ratio on all-cause mortality in patients with type 2 diabetes and foot ulcers: a retrospective cohort study

**DOI:** 10.1186/s12933-022-01534-4

**Published:** 2022-06-03

**Authors:** Jing Hong, Xiang Hu, Wenyue Liu, Xuehua Qian, Feifei Jiang, Zeru Xu, Feixia Shen, Hong Zhu

**Affiliations:** 1 Department of Endocrinology, The First Affiliated Hospital of Wenzhou Medical University, Wenzhou, 325000 China; 2 Department of Information, The First Affiliated Hospital of Wenzhou Medical University, Wenzhou, 325000 China

**Keywords:** Albumin, Diabetes Mellitus, Foot ulcer, Mortality, Inflammation, Red cell distribution width

## Abstract

**Background:**

Red blood cell distribution width (RDW) has emerged as a prognostic factor for mortality in various diseases. Up to now, few studies have focused on the prognostic value of RDW in patients with diabetic foot ulcers (DFUs). This retrospective cohort study aimed to investigate the impact of RDW and RDW/albumin (ALB) ratio on all-cause mortality in patients with DFUs.

**Methods:**

This study included 860 patients with DFUs in a tertiary academic hospital. The associations of RDW and RDW/ALB with all-cause mortality were assessed by multivariable cox regression analyses. The pairwise comparisons of receiver operating characteristic (ROC) curves were performed to compare the predictive performance of RDW and RDW/ALB ratio. Harrell’s concordance index, integrated discrimination improvement, and net reclassification improvement were used to estimate the improvements in risk discrimination.

**Results:**

Patients with high RDW and RDW/ALB had lower overall survival rates (all P < 0.001). The multivariable Cox regression revealed that high RDW [adjusted hazard ratio (HR) 2.426, 95% confidence interval (CI): 1.557–3.778, P < 0.001] and high RDW/ALB (adjusted HR 2.360, 95% CI: 1.414–3.942, P = 0.001) were independent associated with high all-cause mortality. In subgroup analyses, the comparative analysis of ROC curves revealed that the discriminating ability of the RDW/ALB ratio was significantly superior to RDW in patients with no severe DFUs or no severe peripheral artery disease, or in young and middle-aged patients (all P < 0.05). Adding RDW and RDW/ALB ratio to base models improved discrimination and risk reclassification for all-cause mortality.

**Conclusions:**

RDW and RDW/ALB ratio are robust and independent prognostic markers in patients with DFUs. The RDW/ALB ratio appears to be of more predictive value for mortality in younger and less severely ill patients with DFUs. Both RDW and RDW/ALB ratio can provide incremental predictive value for all-cause mortality over traditional risk factors. RDW and RDW/ALB ratio can be used to identify high-risk patients with DFUs.

**Supplementary Information:**

The online version contains supplementary material available at 10.1186/s12933-022-01534-4.

## Introduction

Diabetic foot ulcers (DFUs) are a common and life-threatening complication of diabetes, leading to hospitalization, high health-care costs, and a high rate of amputation [1–3]. DFUs exhibit a 5—year mortality comparable to cancer [2]. Individuals with DFUs have a 2.5-fold increase in the risk for death compared with patients who have diabetes but no DFUs [4]. The excess mortality cannot be fully explained by other known comorbidities and complications of diabetes [4, 5]. It is therefore important to identify and evaluate additional risk factors that influence mortality in patients with DFUs.

The red cell distribution width (RDW) is a simple and easily-obtained parameter, representing the heterogeneity of erythrocyte volume, and is traditionally used for differential diagnosis of anemia [6]. However, in more recent years, RDW was found to be associated with multiple disease processes and prognoses [6]. RDW was associated with a higher risk of developing diabetes [7]. RDW was also associated with diabetes-related complications [8]. RDW can predict mortality and cardiovascular complications in patients with diabetes [9]. However, this observation failed to be corroborated in another population [10], which may be due to the population heterogeneity. RDW/albumin (ALB) ratio is a new combined parameter that can predict mortality in patients undergoing burn surgery, and patients with diabetic ketoacidosis [11, 12]. Diabetes-related complications were associated with an increased inflammatory burden [13, 14]. RDW seems to be a new inflammatory marker. Elevated RDW was found in inflammation-related diseases, such as Hashimoto's thyroiditis [15], thyroid cancer [16], and autoimmune liver diseases [17]. Although a definitive mechanism for the association of RDW, RDW/ALB ratio, and mortality has not yet been established, RDW and RDW/ALB seem to be nonspecific parameters that have the potential to provide effective risk stratification in patients with serious diseases [18].

To date, research related to the prognostic value of RDW with DFUs is scarce [19]. Similarly, there are no previous studies on RDW/ALB ratio in patients with DFUs. In this study, we sought to explore the impact of RDW and RDW/ALB ratio on all-cause mortality in a relatively large cohort of patients with type 2 diabetes and foot ulcers.

## Methods

### Study participants

The study participants were 907 patients diagnosed with type 2 diabetes and DFUs from 2015 to 2019 in the First Affiliated Hospital of Wenzhou Medical University, which is a tertiary academic hospital. The exclusion criteria included terminal malignancies, patients who underwent hemodialysis, or with missing data of RDW or ALB. Ultimately, a total of 860 patients constituted our study population.

The ethics committee of the First Affiliated Hospital of Wenzhou Medical University approved this study. Given the retrospective and non-intrusive nature of the study, the written consent requirement was waived.

### Data collection and grouping

**Fig. 1:**
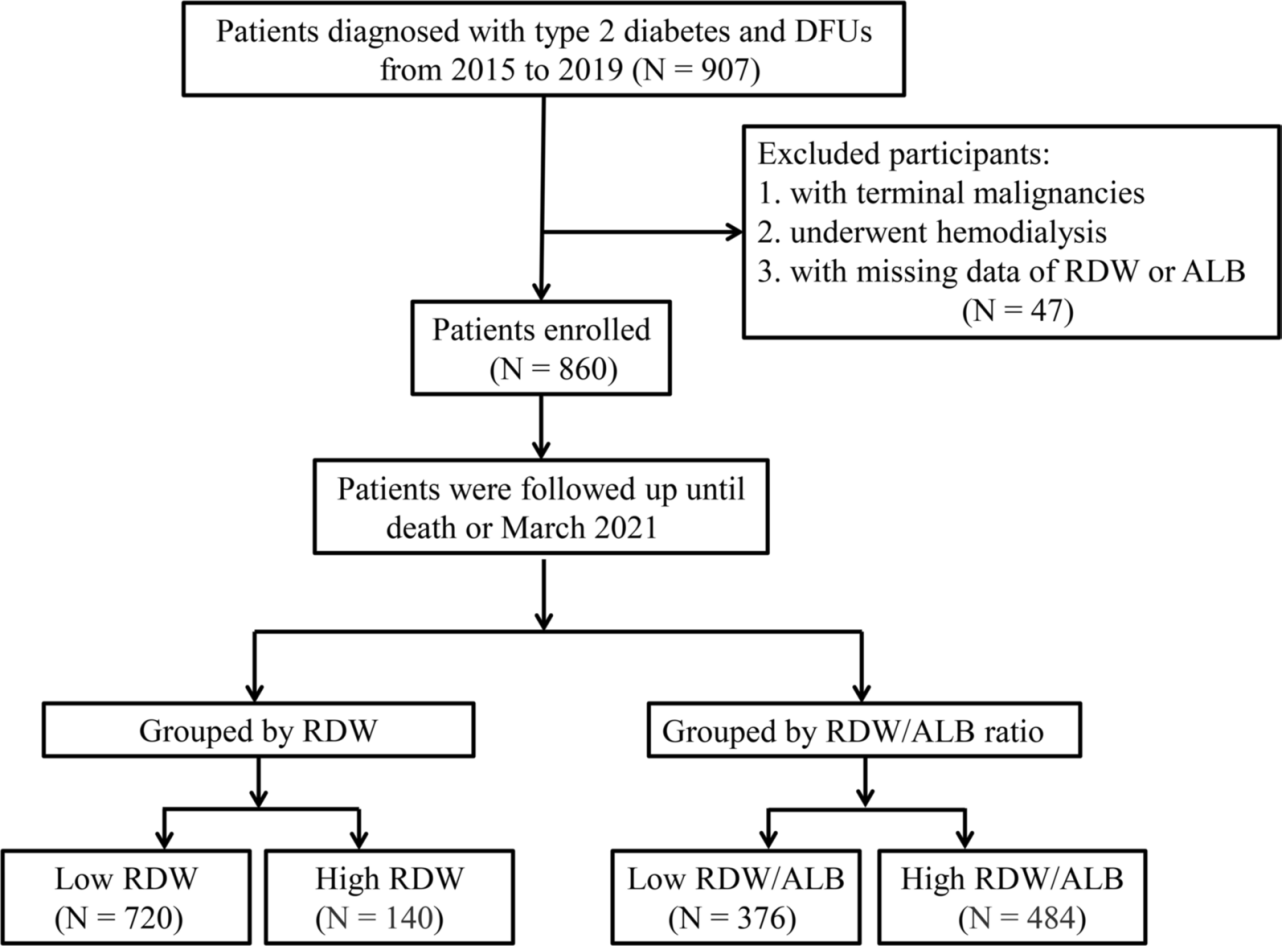
Flow chart of the study. *DFUs* diabetic foot ulcers, *RDW* red cell distribution width, *ALB* albumin

The medical histories and data on baseline characteristics, including demographic, anthropometric, and laboratory parameters, were retrospectively abstracted from individual medical records. For patients with multiple hospitalizations during the study period, only the data of the first hospitalization were included. All-cause mortality was considered as the endpoint. Data regarding deaths was obtained from medical records or by telephone interviews. The follow-up period started at the date of admission and ended at the date of death, or the end of the study (March 2021). The study flow is shown in Fig. 1. Peripheral artery disease (PAD) was diagnosed by ultrasonic diagnostic experts based on the duplex ultrasonography. Severe PAD was defined as the presence of stenosis ≥ 50% in any of the lower extremity arteries. DFUs were defined as the presence of foot ulcer, infection, or deep tissue damage. Severe DFUs were defined as subjects with Wagner grade score ≥ 3 according to the Wagner classification [20]. Coronary artery disease was defined as a previous history of coronary artery disease or a new coronary artery disease diagnosed by cardiologists during hospitalization. Cerebrovascular disease was defined as a history of cerebrovascular disease, or a new cerebrovascular disease diagnosed by computed tomography or magnetic resonance imaging scan during hospitalization. Diabetic retinopathy was diagnosed by digital images of the binocular fundus, if considered necessary by ophthalmologists, further checked by fluorescein fundus angiography and optical coherence tomography. Diabetic peripheral neuropathy was diagnosed by self-reported clinical signs, physical examination, perception thresholds test, and electromyography. Anti-platelet drugs defined as aspirin, clopidogrel or cilostazol. Details of the calculation of estimated glomerular filtration rate (eGFR), definition of hypertension, definition and grouping of the elderly, smoking, alcohol use, severe PAD, and severe DFUs have been described in our previous study [21, 22].

### Statistical analysis

The Kolmogorov–Smirnov test was used for normality tests. All continuous variables were not normally distributed and were expressed as median and interquartile range. Categorical variables were expressed as n (%). Mann–Whitney U test (continuous variables) and Chi-squared test (categorical variables) were used to compare differences between groups. The receiver operating characteristic (ROC) analyses were used to identify the optimal cut-off values of RDW and RDW/ALB ratio for all-cause mortality. To compare the predictive performance of RDW and RDW/ALB ratio, the pairwise comparisons of ROC curves were performed [23]. Kaplan–Meier survival curves with log-rank tests were applied for overall survival (OS) analysis. Unadjusted and multivariable adjusted analyses for all-cause mortality were performed by Cox regression. Variables with P < 0.1 in the unadjusted analysis were selected for the multivariable analyses. ALB was excluded in the analyses of the RDW/ALB ratio. Harrell’s concordance index (C-index), integrated discrimination improvement (IDI), and net reclassification improvement (NRI) were used to estimate the improvements in model performance, discrimination, and risk classification after adding RDW and RDW/ALB to base models (variables with P < 0.1 in the unadjusted Cox regression analysis) [24, 25]. P values < 0.05 were considered statistically significant for all analyses. All statistical analyses were conducted using SPSS (IBM, IL, USA) version 22, MedCalc (MedCalc Software Ltd, Ostend, Belgium) version 20.019, and R version 4.1.2 (R Core Team, survival, survIDINRI, survC1).

## Results

### Patient baseline characteristics

The clinical characteristics of patients categorized based on the cut-off values of RDW and RDW/ALB ratio are summarized in Tables 1 and 2. Patients with high RDW were older, had longer diabetic foot ulcer duration, a greater proportion of severe PAD and anti-hypertensive therapy, and lower eGFR, hemoglobin (Hb), and hemoglobin A1c (HbA1c) than those with low RDW. Patients with high RDW/ALB ratio were older, had longer diabetes duration, greater proportion of severe DFUs and using insulin, lower proportion of anti-platelet therapy, higher HbA1c, and lower body mass index (BMI), diastolic blood pressure (DBP), eGFR, ALB, Hb, total cholesterol (TC), triglyceride (TG), high-density lipoprotein cholesterol (HDL-C), and low-density lipoprotein cholesterol (LDL-C) (all P < 0.05).

**Table 1:** Baseline characteristics of participants with low and high RDW

Characteristic	Low RDW (≤ 14.3%) (N = 720)	High RDW (> 14.3%) (N = 140)	P-value
Male (%)	430 (59.7)	86 (61.4)	0.706
Age (years)	68 (60–76)	73 (65–79)	**0.001**
Age ≥ 65 (%)	454 (63.1)	106 (75.7)	**0.004**
BMI (kg/m^2^)	23.5 (21.6–26.0)	23.5 (21.2–25.8)	0.444
Smoking (%)	213 (29.6)	39 (27.9)	0.681
Alcohol use (%)	180 (25.0)	40 (28.6)	0.376
Diabetes duration (years)	10 (5–18)	10 (6–20)	0.104
Diabetic foot ulcer duration (days)	30 (10–60)	60 (28–98)	** < 0.001**
Severe DFUs (%)	388 (53.9)	81 (57.9)	0.388
PAD			
Severe PAD (%)	370 (59.3)	84 (70.6)	**0.021**
Missing (n)	96	21	
Coronary artery disease (%)	69 (9.6)	21 (15)	0.055
Cerebrovascular disease (%)	63 (8.8)	10 (7.1)	0.532
Diabetic retinopathy (%)	339 (47.8)	58 (41.7)	0.188
Diabetic peripheral neuropathy (%)	370 (51.4)	74 (52.9)	0.750
Hypertension (%)	547 (76.0)	113 (80.7)	0.224
SBP (mmHg)	142 (128–158)	142 (126–160)	0.938
DBP (mmHg)	74 (66–83)	75 (64–84)	0.488
Medication			
Anti-hypertensive drugs (%)	444 (61.7)	99 (70.7)	**0.042**
Two or more anti-hypertensive drugs (%)	200 (27.8)	38 (27.1)	0.878
Insulin (%)	589 (81.8)	109 (77.9)	0.274
Statins (%)	581 (80.7)	120 (85.7)	0.162
Anti-platelet drugs (%)	547 (76.0)	106 (75.7)	0.948
Two or more anti-platelet drugs (%)	162 (22.5)	37 (26.4)	0.313
eGFR (EPI)(mL/min/1.73m^2^)	84.7 (63.2–96.8)	80.5 (50.2–91.4)	**0.003**
ALB (g/L)	34.0 (30.5–37.3)	33.0 (30.3–36.9)	0.205
Hb (g/L)	117 (104–129)	107 (92–122)	** < 0.001**
Fasting glucose (mmol/L)	7.1 (5.3–9.6)	6.3 (4.7–9.4)	**0.029**
HbA1c (%)	8.9 (7.6–10.9)	7.9 (6.8–9.3)	** < 0.001**
TC (mmol/L)	4.04 (3.33–5.02)	3.86 (3.07–4.82)	0.086
TG (mmol/L)	1.26 (0.95–1.78)	1.32 (0.88–1.85)	0.879
HDL-C (mmol/L)	0.91 (0.73–1.11)	0.86 (0.72–1.07)	0.441
LDL-C (mmol/L)	2.29 (1.75–3.00)	2.13 (1.55–2.90)	0.063
RDW (%)	12.9 (12.5–13.4)	15.0 (14.7–15.7)	** < 0.001**
RDW/ALB ratio [%/(g/L)]	0.38 (0.34–0.43)	0.46 (0.41–0.52)	** < 0.001**

*RDW* red cell distribution width, *BMI* body mass index, *DFUs* diabetic foot ulcers, *PAD* peripheral artery disease, *SBP* systolic blood pressure, *DBP* diastolic blood pressure, *eGFR* estimated glomerular filtration rate, *ALB* albumin, *Hb* hemoglobin, *HbA1c* hemoglobin A1c, *TC* total cholesterol, *TG* triglyceride, *HDL* high-density lipoprotein, *LDL* low-density lipoprotein. Results with P value <0.05 were emphasized using bold letters.

**Table 2:** Baseline characteristics of participants with low and high RDW/ALB ratio

Characteristic	Low RDW/ALB (≤ 0.3809) (N = 376)	High RDW/ALB (> 0.3809) (N = 484)	P-value
Male (%)	222 (59.0)	294 (60.7)	0.613
Age (years)	68 (59–76)	70 (62–77)	**0.037**
Age ≥ 65 (%)	235 (62.5)	325 (67.1)	0.156
BMI (kg/m^2^)	23.9 (22.0–26.0)	23.3 (21.4–25.9)	**0.042**
Smoking (%)	117 (31.1)	135 (27.9)	0.303
Alcohol use (%)	104 (27.7)	116 (24.0)	0.218
Diabetes duration (years)	10 (5–16)	10 (6–20)	**0.005**
Diabetic foot ulcer duration (days)	30 (10–60)	30 (15–90)	0.384
Severe DFUs (%)	165 (43.9)	304 (62.8)	** < 0.001**
PAD			
Severe PAD (%)	201 (60.0)	253 (62.0)	0.576
Missing (n)	41	76	
Coronary artery disease (%)	31 (8.2)	59 (12.2)	0.061
Cerebrovascular disease (%)	29 (7.7)	44 (9.1)	0.472
Diabetic retinopathy (%)	167 (44.9)	230 (48.3)	0.321
Diabetic peripheral neuropathy (%)	188 (50.0)	256 (52.9)	0.400
Hypertension (%)	284 (75.5)	376 (77.7)	0.458
SBP (mmHg)	142 (130–157)	142 (125–160)	0.299
DBP (mmHg)	76 (68–83)	73 (65–83)	**0.013**
Medication			
Anti-hypertensive drugs (%)	241 (64.1)	302 (62.4)	0.608
Two or more anti-hypertensive drugs (%)	106 (28.2)	132 (27.3)	0.765
Insulin (%)	286 (76.1)	412 (85.1)	**0.001**
Statins (%)	314 (83.5)	387 (80.0)	0.183
Anti-platelet drugs (%)	303 (80.6)	350 (72.3)	**0.005**
Two or more anti-platelet drugs (%)	101 (26.9)	98 (20.2)	**0.023**
eGFR (EPI)(mL/min/1.73m^2^)	85.9 (68.4–97.1)	81.0 (55.9–95.5)	**0.001**
ALB (g/L)	37.4 (35.5–39.6)	31.2 (27.4–33.2)	** < 0.001**
Hb (g/L)	123 (113–135)	106 (95–121)	** < 0.001**
Fasting glucose (mmol/L)	6.7 (5.4–9.2)	7.1 (5.1–10.1)	0.325
HbA1c (%)	8.3 (7.3–10.0)	8.9 (7.5–11.2)	** < 0.001**
TC (mmol/L)	4.33 (3.36–5.32)	3.80 (3.07–4.62)	** < 0.001**
TG (mmol/L)	1.40 (1.02–1.93)	1.20 (0.87–1.70)	** < 0.001**
HDL-C (mmol/L)	0.91 (0.73–1.11)	0.83 (0.66–1.03)	** < 0.001**
LDL-C (mmol/L)	2.46 (1.84–3.18)	2.13 (1.60–2.80)	** < 0.001**
RDW (%)	12.8 (12.4–13.3)	13.5 (12.8–14.4)	** < 0.001**
RDW/ALB ratio [%/(g/L)]	0.35 (0.33–0.36)	0.44 (0.41–0.50)	** < 0.001**

*RDW* red cell distribution width, *BMI* body mass index, *DFUs* diabetic foot ulcers, *PAD* peripheral artery disease, *SBP* systolic blood pressure, *DBP* diastolic blood pressure, *eGFR* estimated glomerular filtration rate, *ALB* albumin, *Hb* hemoglobin, *HbA1c* hemoglobin A1c, *TC* total cholesterol, *TG* triglyceride, *HDL* high-density lipoprotein, *LDL* low-density lipoprotein. Results with P value <0.05 were emphasized using bold letters.

### Clinical outcomes

**Fig. 2:**
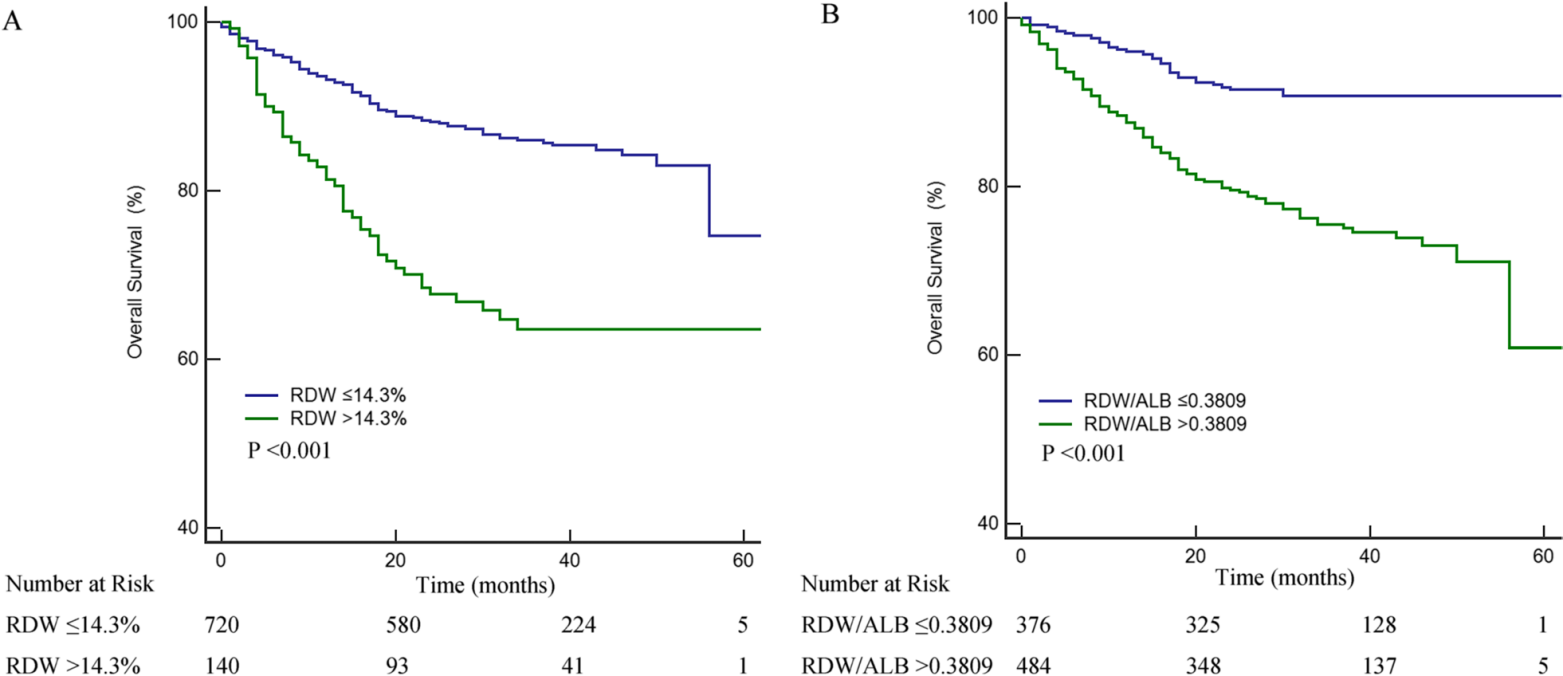
Kaplan–Meier curve of OS. **A** Low RDW (≤ 14.3%) and High RDW (> 14.3%). **B** Low RDW/ALB ratio (≤ 0.3809) and High RDW/ALB ratio (> 0.3809). *OS* Overall Survival, *RDW* red cell distribution width, *ALB *albumin.

During a median follow-up of 32 months, 147 (17.1%) patients died. The Kaplan–Meier curves showed that high RDW and RDW/ALB ratio were related to lower OS rates compared to low RDW and RDW/ALB ratio (all P < 0.001, Fig. 2).

The unadjusted and multivariable-adjusted Cox regression analyses were used to evaluate the prognostic value of RDW and RDW/ALB ratio (Table 3). Variables with P < 0.1 [age, BMI, systolic blood pressure (SBP), severe DFUs, severe PAD, eGFR, ALB, Hb, cerebrovascular disease, diabetic retinopathy and diabetic peripheral neuropathy] in the unadjusted Cox regression analysis (Additional file 2: Table S1), were included as confounding variables in the multivariable cox regression analyses. In the multivariable cox regression analyses, high RDW [adjusted hazard ratio (HR) 2.426, 95% confidence interval (CI): 1.557–3.778, P < 0.001] and high RDW/ALB ratio (adjusted HR 2.360, 95% CI: 1.414–3.942, P = 0.001) were significantly associated with high all-cause mortality. Similar significant robust associations were found in subgroup analyses based on the severity of DFUs and PAD, and in the elderly (≥ 65 years). However, only RDW/ALB was associated with high all-cause mortality in young and middle-aged patients (< 65 years) (Table 3).

**Table 3:** Unadjusted and multivariate cox regression analyses for all-cause mortality

	Unadjusted HR (95% CI)	P-value	Adjusted HR (95% CI)	P-value
Total				
High RDW (vs. Low RDW)	2.802 (1.985–3.957)	** < 0.001**	2.426 (1.557–3.778) ^a^	** < 0.001**
High RDW/ALB (vs. Low RDW/ALB)	2.993 (2.031–4.410)	** < 0.001**	2.360 (1.414–3.942) ^b^	**0.001**
No severe DFUs				
High RDW (vs. Low RDW)	2.780 (1.538–5.023)	**0.001**	2.893 (1.387–6.036) ^c^	**0.005**
High RDW/ALB (vs. Low RDW/ALB)	3.766 (2.006–7.070)	** < 0.001**	3.086 (1.343–7.093) ^d^	**0.008**
Severe DFUs				
High RDW (vs. Low RDW)	2.787 (1.820–4.267)	** < 0.001**	2.539 (1.412–4.563) ^c^	**0.002**
High RDW/ALB (vs. Low RDW/ALB)	2.259 (1.380–3.698)	**0.001**	1.973 (1.026–3.796) ^d^	**0.042**
No severe PAD				
High RDW (vs. Low RDW)	3.940 (1.669–9.305)	**0.002**	4.055 (1.397–11.767) ^e^	**0.010**
High RDW/ALB (vs. Low RDW/ALB)	9.754 (2.286–41.614)	**0.002**	11.585 (1.378–97.397) ^f^	**0.024**
Severe PAD				
High RDW (vs. Low RDW)	2.240 (1.456–3.446)	** < 0.001**	2.461 (1.474–4.107) ^e^	**0.001**
High RDW/ALB (vs. Low RDW/ALB)	2.339 (1.500–3.649)	** < 0.001**	2.028 (1.187–3.463) ^f^	**0.010**
Age < 65				
High RDW (vs. Low RDW)	2.089 (0.693–6.295)	0.191	2.855 (0.678–12.015) ^a^	0.153
High RDW/ALB (vs. Low RDW/ALB)	17.645 (2.362–131.837)	**0.005**	11.217 (1.369–91.925) ^b^	**0.024**
Age ≥ 65				
High RDW (vs. Low RDW)	2.597 (1.801–3.745)	** < 0.001**	2.758 (1.680–4.525) ^a^	** < 0.001**
High RDW/ALB (vs. Low RDW/ALB)	2.485 (1.664–3.711)	** < 0.001**	1.959 (1.143–3.357) ^b^	**0.014**

^a^The multivariable cox regression was adjusted for risk factors including age, BMI, SBP, severe DFUs, severe PAD, Hb, ALB, eGFR, cerebrovascular disease, diabetic retinopathy, diabetic peripheral neuropathy

^b^The multivariable cox regression was adjusted for risk factors including age, BMI, SBP, severe DFUs, severe PAD, Hb, eGFR, cerebrovascular disease, diabetic retinopathy, diabetic peripheral neuropathy

^c^The multivariable cox regression was adjusted for risk factors including age, BMI, SBP, severe PAD, Hb,ALB, eGFR, cerebrovascular disease, diabetic retinopathy, diabetic peripheral neuropathy

^d^The multivariable cox regression was adjusted for risk factors including age, BMI, SBP, severe PAD, Hb, eGFR, cerebrovascular disease, diabetic retinopathy, diabetic peripheral neuropathy

^e^The multivariable cox regression was adjusted for risk factors including age, BMI, SBP, severe DFUs, Hb,ALB, eGFR, cerebrovascular disease, diabetic retinopathy, diabetic peripheral neuropathy

^f^The multivariable cox regression was adjusted for risk factors including age, BMI, SBP, severe DFUs, Hb, eGFR, cerebrovascular disease, diabetic retinopathy, diabetic peripheral neuropathy. In subgroup analyses, all P-value for interactions > 0.05

*RDW* red cell distribution width, *ALB* albumin, *BMI* body mass index, *DFUs* diabetic foot ulcers, *PAD* peripheral artery disease, *SBP* systolic blood pressure, *eGFR* estimated glomerular filtration rate, *Hb* hemoglobin. Results with P value <0.05 were emphasized using bold letters.

### Comparative analysis of ROC curves

**Fig. 3:**
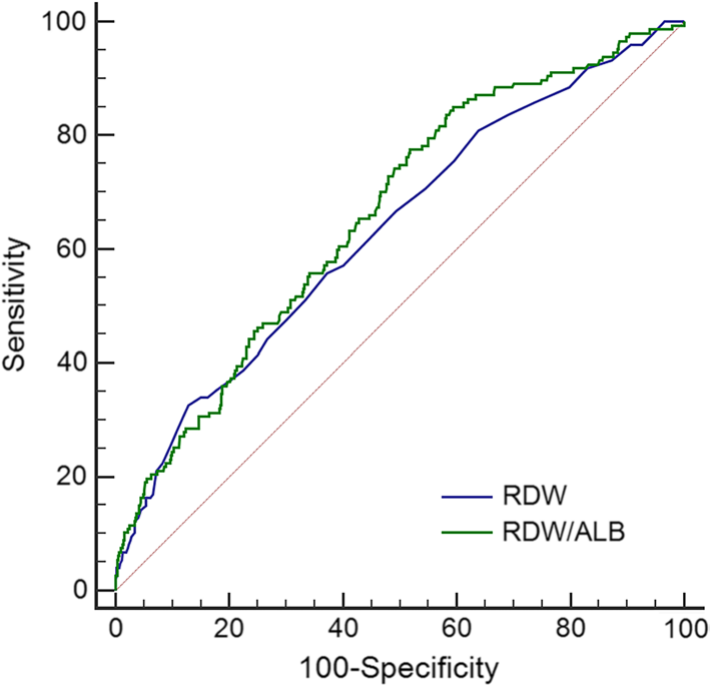
ROC curves of RDW and RDW/ALB ratio for predicting the all-cause mortality in the overall study population. The optimal cut-off values of RDW and RDW/ALB ratio for the all-cause mortality were 14.3% and 0.3809%/(g/L), respectively. The AUC of RDW was 0.634, 95% CI: 0.601–0.666; the AUC of RDW/ALB ratio was 0.660, 95% CI: 0.628–0.692. *ROC* receiver operating characteristic, *RDW* red cell distribution width, *ALB* albumin, *AUC* area under ROC curve

The ROC curves of RDW and RDW/ALB ratio are shown in Fig. 3. According to ROC analyses, the optimal cut-off values of RDW and RDW/ALB ratio were 14.3% and 0.3809%/(g/L), respectively. The sensitivity, specificity, positive predictive value, and negative predictive value of the optimal cut-off values of RDW and RDW/ALB ratio are shown in Additional file 3: Table S2. The comparative analysis of ROC curves revealed that the discriminating ability of the RDW/ALB was significantly superior to RDW: [area under ROC curve (AUC): 0.713, 95% CI: 0.665–0.757 vs. AUC: 0.618, 95% CI: 0.567–0.666, P = 0.025) in patients with no severe DFUs, (AUC: 0.808, 95% CI: 0.758–0.852 vs. AUC: 0.662, 95% CI: 0.604–0.716, P = 0.015) in patients with no severe PAD, and (AUC: 0.741, 95% CI: 0.688–0.790 vs. AUC: 0.551, 95% CI: 0.493–0.609, P = 0.008) in young and middle-aged patients. No significant difference was found between RDW and RDW/ALB ratio by comparative analysis of ROC curves in the overall study population, patients with severe DFUs or severe PAD, or in the elderly (Table 4, Additional file 1: Fig. S1).

**Table 4:** Comparative analysis of ROC curves of RDW and RDW/ALB ratio for all-cause mortality

	RDW AUC (95% CI)	RDW/ALB AUC (95% CI)	DBA (95%CI)	P-value
Total	0.634 (0.601–0.666)	0.660 (0.628–0.692)	0.027 (− 0.027 to 0.080)	0.329
No severe DFUs	0.618 (0.567–0.666)	0.713 (0.665–0.757)	0.095 (0.012 to 0.179)	**0.025**
Severe DFUs	0.650 (0.605–0.693)	0.606 (0.561–0.651)	0.044 (− 0.026 to 0.113)	0.216
No severe PAD	0.662 (0.604–0.716)	0.808 (0.758–0.852)	0.146 (0.028 to 0.264)	**0.015**
Severe PAD	0.610 (0.564–0.656)	0.630 (0.583–0.674)	0.019 (− 0.046 to 0.084)	0.562
Age < 65	0.551 (0.493–0.609)	0.741 (0.688–0.790)	0.190 (0.050 to 0.330)	**0.008**
Age ≥ 65	0.625 (0.583–0.665)	0.654 (0.613–0.693)	0.029 (− 0.029 to 0.087)	0.329

*ROC* receiver operating characteristic, *RDW* red cell distribution width, *ALB* albumin, *DBA* difference between areas, *AUC* area under curve, *DFUs* diabetic foot ulcers, *PAD* peripheral artery disease. Results with P value <0.05 were emphasized using bold letters.

### Incremental predictive value of RDW and RDW/ALB ratio

Adding RDW and RDW/ALB ratio to base models significantly improved the predictive ability for all-cause mortality. RDW increased the C-index from 0.792 to 0.804 and RDW/ALB ratio increased the C-index from 0.783 to 0.794. IDI showed a significant improvement of 2.7% (95% CI: 0.2–6.3%, P = 0.02) and 2.3% (95% CI: 0.4–5.5%, P = 0.02) after adding RDW and RDW/ALB ratio to base model, respectively. NRI was also significant for RDW (23.4%, 95% CI: 3.0–30.8%, P = 0.03) and RDW/ALB ratio (29.6%, 95% CI: 11.0–40.0%, P = 0.02) (Table 5).

**Table 5:** Improvement in discrimination and risk reclassification for all-cause mortality after the addition of RDW or RDW/ALB

Model	C-index (95% CI)	IDI (%) (95% CI)	P-value	NRI (%) (95% CI)	P-value
Base model 1	0.792 (0.745–0.839)	Ref	Ref	Ref	Ref
Base model 1 + RDW	0.804 (0.759–0.849)	2.7 (0.2–6.3)	**0.02**	23.4 (3.0–30.8)	**0.03**
Base model 2	0.783 (0.736–0.830)	Ref	Ref	Ref	Ref
Base model 2 + RDW/ALB	0.794 (0.747–0.841)	2.3 (0.4–5.5)	**0.02**	29.6 (11.0–40.0)	**0.02**

IDI and NRI were calculated at 32 months. Base model 1, adjusted for factors including age, BMI, SBP, severe DFUs, severe PAD, Hb, ALB, eGFR, cerebrovascular disease, diabetic retinopathy, diabetic peripheral neuropathy. Base model 2, adjusted for risk factors including age, BMI, SBP, severe DFUs, severe PAD, Hb, eGFR, cerebrovascular disease, diabetic retinopathy, diabetic peripheral neuropathy.

*RDW* red cell distribution width, *ALB* albumin, *C-index* Harrell’s concordance index, *IDI* integrated discrimination improvement, *NRI* net reclassification improvement index, *Ref* reference. Results with P value <0.05 were emphasized using bold letters.

## Discussion

In this retrospective cohort study, both high RDW and RDW/ALB ratio at the time of admission were associated with higher all-cause mortality in a cohort of 860 patients with DFUs treated in a tertiary academic hospital. The risk of mortality associated with high RDW and RDW/ALB ratio remained statistically significant even after adjustment for confounding variables. In subgroup analyses, the comparative analysis of ROC curves showed that the discriminating ability of the RDW/ALB was significantly superior to RDW in patients with no severe DFUs or no severe PAD, or young and middle-aged patients. Additionally, both RDW and RDW/ALB ratio significantly improved predictive ability for all-cause mortality over traditional risk factors. To the best of our knowledge, this is the first study to investigate the predictive value of RDW/ALB ratio, a new combined biomarker, together with RDW in patients with DFUs.

Only few studies have focused so far on the RDW in patients with DFUs. A previous study with a small sample size in Turkey reported that RDW was a predictive parameter for major amputation in patients with DFUs [26]. A recent conference abstract with little available detail indicated that RDW was a risk factor for all-cause mortality in a moderate-sized cohort of patients undergoing amputations due to DFUs [19]. No earlier studies have reported the association between RDW/ALB ratio and all-cause mortality in patients with DFUs. This study demonstrated the predictive value of RDW and RDW/ALB ratio concurrently in a relativity large cohort of patients with DFUs.

The underlying mechanisms of the associations between high RDW and mortality have not been fully elucidated. RDW may be considered as a general marker of health status, rather than disease-specific, which is associated with mortality in a variety of diseases, as well as in the general population [6]. There are several reasons leading to the higher RDW, including inflammation [27], oxidative stress [28], shortening of telomeres length [29], increased erythrocyte mechanical fragility [30], nutritional deficiencies [31], and deficiency or dysfunction of erythropoietin [6]. All of the aforementioned conditions were important prognostic factors for mortality [6]. Inflammation contributes to higher RDW by myelosuppression, promoting red cell apoptosis and erythropoietin resistance, reducing erythropoietin production and bioavailability of iron [32–35]. Oxidative stress induces increased RDW by shortening the life span of erythrocytes and increasing the migration of premature erythrocytes to the peripheral circulation [36]. Shortening of telomeres length is a telltale sign of cellular aging and is associated with age-related diseases, including diabetes [37]. Shortening of telomeres length causes cell senescence of the erythromyeloid progenitors, thus leading to an impaired capacity of replicative and maturation of erythrocytes [38]. Diabetic nephropathy is associated with erythrocyte fragmentation, and renal dysfunction is often accompanied by deficiency of erythropoietin [6, 39], thus leading to increased RDW [40]. Patients with high RDW in the present study were older, had lower eGFR, HB, BMI. However, the associations of RDW and RDW/ALB ratio with all-cause mortality remained significant after adjustment for age, eGFR, Hb, and BMI. Hence, the pathophysiological mechanisms for the predictive roles of RDW in patients with DFUs can not be fully explained by the aforementioned conditions, and need to be elucidated by further studies.

RDW is routinely measured as part of the extensively used complete blood counts. Hence, it would not require any additional cost. RDW also seems to be potentially modifiable. High RDW can be lowered by exercise training in patients with coronary artery disease [41], and by treatment with iron in hemodialysis patients [42]. However, further studies are warranted to clarify whether aggressive interventions on RDW can improve the outcomes of patients with high RDW. Moreover, due to the characteristics of patients with DFUs, with limited mobility, interventions on RDW might differ from the aforementioned patients.

In this study, the discriminating ability of RDW/ALB for all-cause mortality was significantly superior to RDW in younger, healthier, and less severely ill patients by comparative analysis of ROC curves. The ROC curves analysis also indicated that RDW/ALB ratio had better predictive power for mortality than RDW in patients undergoing burn surgery and with acute respiratory distress syndrome [12, 43]. ALB was widely applied to assess the nutritional status and reflect the systemic inflammation [44]. Hypoalbuminemia was associated with mortality in various diseases and healthy individuals [45]. The combination of RDW and ALB may be more strongly associated with mortality than a single indicator in a particular clinical situation.

The key strengths of this study include the relatively large sample size and the use of widely available and inexpensive parameters: RDW and RDW/ALB ratio, which can be used in a variety of clinical settings, even in some economically underdeveloped areas. However, there are still several limitations that should be considered. First, our analysis is restricted to all-cause mortality, not cause-specific mortality. However, it was considered to be difficult and subjective in classifying the cause of death in patients who have multiple problems, and the all-cause mortality was an objective and clinically useful endpoint [46]. Second, RDW and RDW/ALB ratio were assessed only at one-time point at baseline, and changes over time were not accounted for in this study. Finally, this is a hospital-based study conducted in a single tertiary academic hospital with patients having greater disease burden, hence, the results might not apply to other populations. Further studies in different settings and cohorts with dynamic observations of RDW and RDW/ALB ratio are needed to clarify the predictive value of increased RDW and RDW/ALB ratio.

## Conclusion

The most important conclusions of this study are the following: In patients with DFUs, RDW and RDW/ALB ratio are independent prognostic markers for all-cause mortality. The discriminating ability of the RDW/ALB ratio for all-cause mortality was significantly superior to RDW in patients with no severe DFUs or no severe PAD, or young and middle-aged patients. The combination of RDW and ALB might give a more efficacious approach for the assessment of mortality in patients with mild DFUs. All in all, RDW and RDW/ALB ratio are simple and practical parameters that may be useful in risk stratification of patients with DFUs, sequentially improving outcomes of those high-risk patients by intensive management, which needs to be confirmed by further studies.

## Supplementary Information

**Additional file 1: Fig. S1**. ROC curves of RDW and RDW/ALB ratio for predicting the all-cause mortality in: (A) patients with no severe DFUs, (B) patients with severe DFUs, (C) patients with no severe PAD, (D) patients with severe PAD, (E) young and middle-aged patients, and (F) the elderly. The discriminating ability of the RDW/ALB ratio was superior to RDW in patients with (A) no severe DFUs, (C) no severe PAD, or in (E) young and middle-aged patients (all P < 0.05). ROC: receiver operating characteristic; RDW: red cell distribution width; ALB: albumin; DFUs: diabetic foot ulcers; PAD: peripheral artery disease.

**Additional file 2: Table S1**. Unadjusted Cox regression analyses for all-cause mortality.

**Additional file 3: Table S2**. Diagnostic performances of optimal cut-off values of RDW and RDW/ALB ratio.

## Data Availability

The datasets used and analyzed during the current study are available from the corresponding author on reasonable request.

## Abbreviations

ALB: Albumin
AUC: Area under ROC curve
BMI: Body mass index
C-index: Harrell’s concordance index
CI: Confidence interval
DBA: Difference between areas
DBP: Diastolic blood pressure
DFUs: Diabetic foot ulcers
eGFR: Estimated glomerular filtration rate
HbA1c: Hemoglobin A1c
Hb: Hemoglobin
HDL: High-density lipoprotein
HR: Hazard ratio
IDI: Integrated discrimination improvement
LDL: Low-density lipoprotein
NPV: Negative predictive value
NRI: Net reclassification improvement index
OS: Overall survival
PAD: Peripheral artery disease
PPV: Positive predictive value
Ref: Reference
ROC: Receiver operating characteristic
SBP: Systolic blood pressure
TC: Total cholesterol
TG: Triglyceride
