# Supplementary figures and images for: Impact of red cell distribution width and red cell distribution width/albumin ratio on all-cause mortality in patients with type 2 diabetes and foot ulcers: a retrospective cohort study
Source: Cardiovasc Diabetol. 2022 Jun 3;21:91. doi: 10.1186/s12933-022-01534-4 (PMC9166463; doi:10.1186/s12933-022-01534-4)

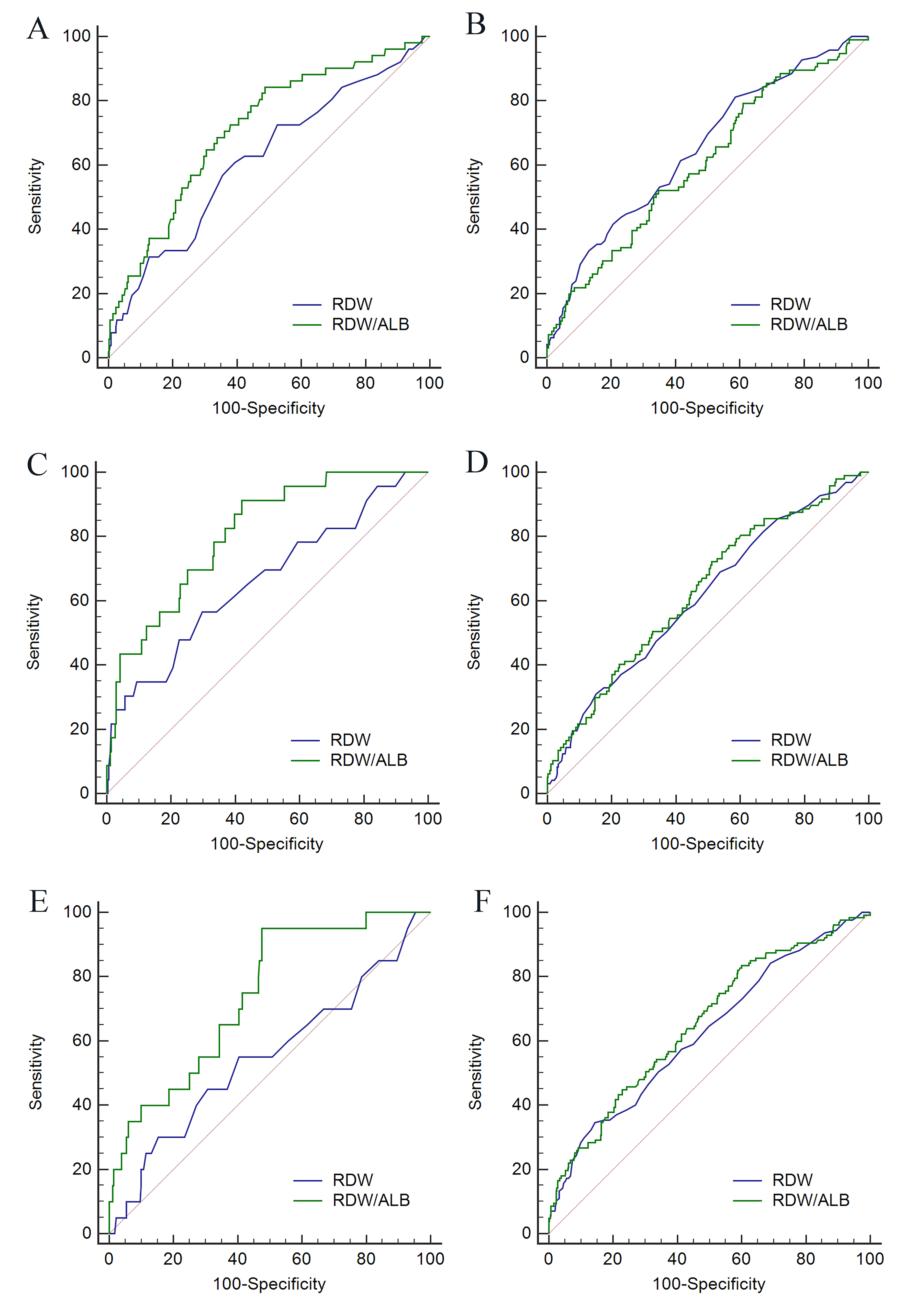

Supplement: Supplementary file 1 — Additional file 1: Fig. S1. ROC curves of RDW and RDW/ALB ratio for predicting the all-cause mortality in: (A) patients with no severe DFUs, (B) patients with severe DFUs, (C) patients with no severe PAD, (D) patients with severe PAD, (E) young and middle-aged patients, and (F) the elderly. The discriminating ability of the RDW/ALB ratio was superior to RDW in patients with (A) no severe DFUs, (C) no severe PAD, or in (E) young and middle-aged patients (all P < 0.05). ROC: receiver operating characteristic; RDW: red cell distribution width; ALB: albumin; DFUs: diabetic foot ulcers; PAD: peripheral artery disease. [file 12933_2022_1534_MOESM1_ESM.tif]
