# Supplementary material for: Impact of red cell distribution width and red cell distribution width/albumin ratio on all-cause mortality in patients with type 2 diabetes and foot ulcers: a retrospective cohort study
Source: Cardiovasc Diabetol. 2022 Jun 3;21:91. doi: 10.1186/s12933-022-01534-4 (PMC9166463; doi:10.1186/s12933-022-01534-4)
Supplement: Supplementary file 2 — Additional file 2: Table S1. Unadjusted Cox regression analyses for all-cause mortality. [file 12933_2022_1534_MOESM2_ESM.docx]

Supplemental Table 1 Unadjusted Cox regression analyses for all-cause mortality

| Characteristic | Unadjusted HR (95%CI) | P-value |
| --- | --- | --- |
| Male (vs. Female) | 1.024 (0.737-1.424) | 0.887 |
| Age (per year increase) | 1.078 (1.059-1.097) | **< 0.001** |
| BMI (per 1-kg/m^2^ increase) | 0.941 (0.888-0.998) | **0.041** |
| Smoking (vs. non-smoking) | 0.825 (0.570-1.194) | 0.308 |
| Alcohol use (vs. no alcohol consumption) | 0.809 (0.547-1.197) | 0.288 |
| Diabetes duration (per year increase) | 1.000 (0.984-1.016) | 0.981 |
| Diabetic foot duration (per day increase) | 1.000 (1.000-1.001) | 0.602 |
| Severe DFUs (vs. no severe DFUs) | 1.777 (1.264-2.498) | **0.001** |
| Severe PAD (vs. no severe PAD) | 1.803 (1.416-2.296) | **< 0.001** |
| Coronary artery disease (vs. no coronary artery disease) | 1.212 (0.740-1.985) | 0.445 |
| Cerebrovascular disease (vs. no cerebrovascular disease) | 1.667 (1.029-2.699) | **0.038** |
| Diabetic retinopathy (vs. no diabetic retinopathy) | 0.576 (0.410-0.809) | **0.001** |
| Diabetic peripheral neuropathy  (vs. no diabetic peripheral neuropathy) | 0.640 (0.461-0.888) | **0.008** |
| Hypertension (vs. no hypertension) | 1.079 (0.732-1.590) | 0.701 |
| SBP (per 1-mmHg increase) | 1.006 (0.999-1.013) | 0.075 |
| DBP (per 1-mmHg increase) | 0.990 (0.977-1.003) | 0.127 |
| Anti-hypertensive drugs (vs. no anti-hypertensive drugs) | 0.933 (0.668-1.303) | 0.685 |
| Two or more anti-hypertensive drugs  (vs. less than two anti-hypertensive drugs) | 1.269 (0.899-1.791) | 0.176 |
| Insulin (vs. no insulin) | 0.812 (0.551-1.197) | 0.292 |
| Statins (vs. no statins) | 1.183 (0.757-1.850) | 0.460 |
| Anti-platelet drugs (vs. no anti-platelet drugs) | 0.985 (0.673-1.440) | 0.936 |
| Two or more anti-platelet drugs  (vs. less than two anti-platelet drugs) | 1.152 (0.797-1.667) | 0.452 |
| eGFR (EPI) (per 1-mL/min/1.73m^2^ increase) | 0.984 (0.978-0.990) | **< 0.001** |
| ALB (per 1-g/L increase) | 0.935 (0.909-0.962) | **< 0.001** |
| Hb (per 1-g/L increase) | 0.975 (0.967-0.983) | **< 0.001** |
| HbA1c (per 1% increase) | 0.957 (0.879-1.042) | 0.315 |
| TC (per 1-mmol/L increase) | 0.954 (0.847-1.074) | 0.438 |
| TG (per 1-mmol/L increase) | 0.946 (0.801-1.117) | 0.514 |
| HDL-C (per1-mmol/L increase) | 0.702 (0.412-1.197) | 0.194 |
| LDL-C (per1-mmol/L increase) | 0.994 (0.842-1.173) | 0.945 |
| RDW (%) (per 1% increase) | 1.239 (1.160-1.323) | **< 0.001** |
| RDW/ALB ratio [per 1-%/(g/L)increase] | 103.024 (29.613-358.421) | **< 0.001** |

RDW: red cell distribution width; BMI: body mass index; DFUs: diabetic foot ulcers; PAD: peripheral artery disease; SBP: systolic blood pressure; DBP: diastolic blood pressure; eGFR: estimated glomerular filtration rate; ALB: albumin; Hb: hemoglobin; HbA1c: hemoglobin A1c; TC: total cholesterol; TG: triglyceride; HDL: high-density lipoprotein; LDL: low-density lipoprotein.
