# Supplementary material for: Impact of red cell distribution width and red cell distribution width/albumin ratio on all-cause mortality in patients with type 2 diabetes and foot ulcers: a retrospective cohort study
Source: Cardiovasc Diabetol. 2022 Jun 3;21:91. doi: 10.1186/s12933-022-01534-4 (PMC9166463; doi:10.1186/s12933-022-01534-4)
Supplement: Supplementary file 3 — Additional file 3: Table S2. Diagnostic performances of optimal cut-off values of RDW and RDW/ALB ratio. [file 12933_2022_1534_MOESM3_ESM.docx]

Supplemental Table 2 Diagnostic performances of optimal cut-off values of RDW and RDW/ALB ratio

| Cut-off values | Sensitivity (%) | Specificity (%) | PPV (%) | NPV (%) |
| --- | --- | --- | --- | --- |
| RDW: 14.3% | 32.7 | 87.1 | 34.3 | 86.2 |
| RDW/ALB: 0.3809 [%/( g/L)] | 77.6 | 48.1 | 23.6 | 91.2 |

RDW: red cell distribution width; ALB: albumin; PPV: positive predictive value; NPV: negative predictive value.
